# Statistical processing of building and neighborhood data considering energy ratings in Dublin, Ireland

**DOI:** 10.1016/j.dib.2024.110954

**Published:** 2024-09-21

**Authors:** Nasim Eslamirad, Mehdi Gholamnia, Payam Sajadi, Francesco Pilla

**Affiliations:** School of Architecture Planning and Environmental Policy, University College Dublin, Ireland

**Keywords:** Spatial Data, Built environment analysis, City energy analysis, Building energy rating

## Abstract

This paper presents a methodology aimed to enhance urban energy analysis through the utilization of geospatial data to collect and integrate not only building data but also data related to the urban context in which buildings are situated. Utilizing datasets like the GeoDirectory Building Energy Ratings (BER) dataset of Ireland, supplemented by data of Digital Landscape Models (DLM) Core Data from Tailte Éireann Surveying (PRIME2 Dataset), landscape map of Dublin, we acquire both geometric and non-geometric data related to buildings in Dublin at both building and neighborhood scales. These datasets enable us to perform effective neighborhood-scale analysis and built environment analysis within a geospatial context. Our methodology employs a diverse array of tools and software, including programming languages such as MATLAB and Python ( in the Jupyter Notebook interface), with libraries such as Geopandas, Pandas, NumPy, Seaborn, and Scikit-learn were used for data processing and analysing. In addition, we conduct geospatial analyses using the toolbox and plugins of the ArcGIS and QGIS software. Our data integration encompasses various parameters including building attributes, neighborhood characteristics, and urban-scale built environment metrics at both building and neighborhood scales. This comprehensive dataset provides valuable insights into building energy performance and urban energy dynamics. Researchers can leverage this data to develop data-driven approaches and predictive models for analyzing environmental factors, thereby formulating effective urban planning strategies for sustainability and energy analysis of buildings, neighborhoods, and residential zones in Dublin.

Specifications Table

**Table:** 

Subject	Engineering, Architecture
Specific subject area	Utilizing Architectural and Urban Analysis for Evaluating Building Energy
Type of data	Shapefiles, Vector dataset, csv files Analyzed and Processed data
Data collection	Central to our investigation is the Building Energy Rate (BER) dataset [1] of Ireland, sourced from GeoDirectory data, which provides a foundational resource. This dataset includes a comprehensive range of building-scale parameters, such as non-geometric features like address, geocode data, area, height, number of floors, roof type, construction age, radon emission, HVAC system details, and bedroom and bathroom counts. In addition, the dataset includes some geometric features such as building height, area, small area. Moreover, additional features were obtained through Digital Landscape Models (DLM) Core Data from Tailte Éireann Surveying (PRIME2 Dataset) [2], including processing and calculations based on the landscape map of Dublin. These encompass metrics like density of the built environment surrounding each building, and district attributes like the ratio of green area to non-green area in the urban vicinity.
Data source location	Data Storage Address Institution: University College Dublin City/Town/Region: Dublin Country: Ireland Latitude and longitude (and GPS coordinates, if possible) for collected samples/data: 53.5°N and 6.5°W
Data accessibility	Repository name: MendeleyData, Reserved DOI: 10.17632/8yvy2g2zxs.4 Direct URL to dataset, Figures, and Tables: https://data.mendeley.com/datasets/kvbgzr6dn8/1 Direct URL to codes: https://github.com/MehdiGhUCD/Geospatial-Analysis-of-Residential-Buildings-
Related research article	No research article is related to the data preparation.

## 1 Value of the Data

• Unique dataset for Ireland: This dataset fills a gap for Ireland, providing crucial buildings data that can be used to develop data-driven approaches and create explainable and predictive models. Existing datasets often lack detailed information about the neighborhood context of buildings. By integrating environmental factors at both the building and city scales, this dataset supports more effective urban planning strategies aimed at sustainability.
• Generalizable and scalable methodology: The methodology used to generatethis dataset is generalizable and can be applied to develop similar datasets for other locations and cities. Since most urban areas have comparable datasets, this approach can be widely adopted. Given that most urban areas possess comparable datasets, this approach can be widely implemented. The scalable and adaptable computational methods used in this research equip urban planners and policymakers with powerful tools to make informed, data-driven decisions, ultimately fostering sustainable urban development.
• Comprehensive insights: The dataset provides detailed insights into buildings and their energy performance by considering individual building features as well as their context within the built environment and land cover. This dual focus enhances the understanding of energy dynamics at various scales. As a result, the dataset can be utilized in a wide range of research studies, including those focused on evaluating building energy performance, urban energy retrofitting, and the broader energy efficiency of urban areas.
• Enhanced understanding of urban energy dynamics: By including crucial information on the spatial characteristics of buildings and their surroundings, the dataset enables a deeper understanding of urban energy dynamics. This is vital for addressing energy efficiency and sustainability in urban areas. For instance, in predictive models of urban energy use, this dataset allows for the inclusion of both building-specific and neighborhood-level data, alongside the energy rating of each building unit.Diverse features: The dataset includes a wide range of features such as building specifications, neighborhood attributes, land coverage, and urban-scale built environment metrics. These diverse features increase the dataset's applicability across various research domains and contribute to a more comprehensive understanding of urban energy dynamics.
• Stakeholders and potential beneficiaries: Urban planners, policymakers, researchers, and sustainability advocates are among the primary stakeholders who can benefit from this dataset. It provides them with the data necessary to drive informed decisions and strategies in urban energy management, sustainability planning, and policy development.

## 2 Background

With Ireland's population poised for significant expansion, particularly in urban areas, prioritizing quality in new residential developments becomes imperative for fostering sustainable communities [3]. Moreover, this growth poses challenges, as the building and construction sector accounted for 39 % of process-related carbon dioxide emissions in 2018 [4]. Therefore, improving the energy efficiency of buildings is pivotal to the European Union's efforts to meet its energy and climate targets. Achieving a comprehensive understanding of buildings and urban area energy demand, needs, and consumption is essential. A thorough understanding of comprehensive urban data requires detailed information about buildings and their context within the built environment. Filip Biljecki and Yoong Shin Chow [4] conducted a comprehensive study on building data across multiple scales—both at the individual building level and within its broader contextual setting. Their research significantly contributes to urban analytics and supports a wide range of related disciplines. This recognition has prompted us to explore additional factors that influence building energy consumption beyond basic geometric and structural attributes. While the BER system offers a standardized assessment of energy efficiency, ranging from A1 to G, it may not fully capture the intricate relationship between building features and the surrounding built environment. Therefore, it's vital to incorporate features such as built environment density, land cover types, building distances, and orientations into the dataset. These neighborhood-scale attributes can provide valuable insights into the energy profiles of buildings within a cityscape.

### 2.1 The Data Acquisition Process Involves Two Main Scales

• Building scale: The data collection process utilizes the GeoDirectory database, offering comprehensive insights into individual buildings. This dataset not only provides detailed building information but also incorporates geospatial features. In addition, supplementary features pertinent to each building were derived through calculation and analysis methods.
• Neighbourhood scale: Geospatial data capture and analysis are conducted using the GeoDirectory and Tailte Éireann Surveying Datasets. These datasets are utilized for various purposes, including direct data usage, processing and calculations, as well as analysis and reproduction of data related to residential buildings in Dublin. The GeoDirectory BER dataset provides both geometric and non-geometric data at the individual building scale. This includes detailed information such as building dimensions, construction age, and HVAC system details. Additionally, metrics related to the urban area analysis at the neighborhood scale, such as the nearest neighbor building to each individual building sample, are derived from the analysis of the GeoDirectory dataset. Furthermore, the DLM Core Data of Tailte Éireann Surveying (the PRIME2 Dataset) are utilized for analyzing the surrounding urban environment. This includes conducting geoprocessing and analysis to derive metrics such as the density of the built environment surrounding each building. Additionally, land cover analysis of the area surrounding each building's geometry is performed using these datasets.

## 3 Data Description

The data resources encompass two distinct resources: GeoDirectory and Tailte Éireann. Through a combination of data collection and analysis, datawas gathered at two distinct scales: building and neighborhood. These datasets were instrumental in organizing the data into three distinct groups, providing a comprehensive understanding of both individual buildings and the surrounding urban context.

• Building- scale data, related to individual buildings that primarily originates from the GeoDirectory BER dataset.
  ○ Building Address References: ID, Geographic Coordinates, Official Postal Format
  ○ Building Geocode Data: Latitude and Longitude (Lat/Log)
  ○ Building Features: Usage, Type, Construction, Age, Number of Floors, Height, Area, Orientation
  ○ Building Energy Resources and HVAC System Data
  ○ Building Radon Emission
  ○ Building Sales Information
  ○ Building Energy Rating (BER)
• Neighborhood- scale data, related to geospatial data capturing and analysis of built environment in the neighborhood scale surrounding each building using the map of DLM Core Data, Tailte Éireann Surveying (the PRIME2 Dataset).
  ○ Density of the built environment (in 100 m radius from the center of each building)
  ○ Land cover type of the built environment (whitin 100 m radius from the center of each building center)

### 3.1 Geospatial BER Dataset

Energy modelling of the built environment requires both geometric and non-geometric data [5]. This study integrates real building geometric data, encompassing features and volume, with non-geometric data such as building type, roof type, HVAC systems, Radon emissions, and energy ratings sourced from the GeoDirectory BER dataset of Ireland as a single format flat CSV geospatial and Shape file [5].

Table 1 shows the description of data that are provided in the GeoDirectory dataset.

**Table 1:** Data provided in the Geodirectory dataset [4].

	Column names	Columns size and type	Columns description
Building address references	ADDRESS_REFERENCE	VARCHAR2(18 BYTE)	Unique reference for every address in Ireland, Combination of the Building-id and the Address-Point_ID. If the Address_Point_ID is null then the Address-References is padded with zero's.
	BUILDING_ID	NUMBER (8,0)	Unique 8digit identification number for the building.
	ADDRESS_POINT-ID	NUMBER (8,0)	Unique 8digit identification number for the Address Points (sub-building units)
	ADDR_LINE_1…10	VARCHAR2(200 BYTE)	Standardized address in Fields ADDR_Line-1-ADD_LINE_10.
	POSTAL_ADDR_LINE_1…10	VARCHAR2(200 BYTE)	The official Postal address in fields POSTAL_ADDR_LINE_1-POSTAL_ADDR_LINE_10.
	CUNTRY_NAME	VARCHAR2(40 BYTE)	The name of the country the building is in.
	EIRCODE	VARCHAR2(7 BYTE)	The Eircode associated with the address.
Building geocode data	LATITUDE	VARCHAR2(100 BYTE)	Geocode of the Building expresses as a Latitude.
	LONGITUDE	VARCHAR2(100 BYTE)	Geocode of the Building expresses as a Longitude.
Building features	BUILDING_HEIGHT^1^	NUMBER (10, 2)	Height of the building above ground level in meters.
	GROUND_HEIGHT	NUMBER (10, 2)	Height of the building above Mean Sea Level.
	AREA (SQ, METER)^2^	NUMBER (10, 2)	The area of the building in square meters.
	FLOORS^3^	NUMBER (3)	The number of floors in the building.
	SMALL_AREA	VARCHAR2(100 BYTE)	The small areas associated with a building.
	YEAR_OF_BUILD^4^	VARCHAR2(100 BYTE)	Year or range of build year
	BUILDING_USE	VARCHAR2(1 BYTE)	Building use column indicates whether this building is residential (R), commercial (C), both residential and commercial (B) or unknown (U)
	BUILDING_TYPE_NAME	VARCHAR2(50 BYTE)	The Types of Building e.g. Bungalow, Detached etc.
Building sales information	PROPERTY_SALE_PRICE	NUMBER (30, 2)	The value of sales price of the property in euros.
	DATE_OF_SALE	DATE	The actual date of property sold.
BER data	BER_ENERGY_MEDIAN^5^	NUMBER (30, 2)	The median energy rating expressed in KWh/m^2^ /year for small area.
	BER_ENERGY_HIGH	VARCHAR2(100 BYTE)	Percentage of buildings in the range from A1-C3.
	BER_ENERGY_LOW	VARCHAR2(100 BYTE)	Percentage of buildings in the range from D1-G.
	BER_ENERGY_MODE	VARCHAR2(2 BYTE)	BER rating that appears most often in the small area.

1 Source: Ordnance survey Ireland (OSi), Building height extracted from their Lidar dataset to estimate the height of buildings.

2 Source: Ordnance survey Ireland (OSi), Building footprint (area)extracted from PRIME 2 data set (latest surveyed building polygons).

3 Based on the number of windows or part of windows we see above ground level.

4 Source: GeoDirectory, The year from GeoDirectory if post 2002. Some heritage buildings sourced externally. Reminder expressed as a range. The highest probability method, Small Area and CSO 2016 Census data, 10 or 30 year bands, Pre 1945 are sorted as 1900-1945.

5 Source: Sustainable Energy Ireland (SEI). Each value of BER is derived using Small Areas and SEI data. The values are assigned to each building in the Small Area.

### 3.2 BER Dataset Statistic

The statistical summary in Table 2 provides insights into various features of the BER dataset, covering geometrical, non-geometrical, and categorical aspects, while excluding location-based details. It includes important attributes such as building type, BER rating mode, year of build, bedroom and bathroom estimates, heating fuel type, predominant roof type, and radon risk, along with the count of each class for a comprehensive understanding of data distribution and variability.

**Table 2:** Statistics of the BER dataset, categorical features.

Feature	Category	Count
BUILDING_TYPE_NAME	TERRACED	67714
	SEMI-DETACHED	32324
	DETACHED	10832
	BUNGALOW	2094
	DUPLEX	332
	TEMPORARY STRUCTURE	10
BER_RATING_MODE	G	36606
	E1	27723
	F	25799
	E2	23178
YEAR_OF_BUILD	<1899	26315
	1946–1960	23561
	1900–1945	16545
	1971–1980	16272
	1961–1970	11205
	1981–1990	5894
	1991–2000	3524
	2000–2002	2649
	2017	1086
EST_BEDROOMS	2	3743
	3	7653
	4	5
EST_BATHROOMS	1	37274
	2	7780
EST_WATER_HEATING_FUEL	Mains Gas	98125
	Electricity	4712
	Heating Oil	2282
	Solid Multi-Fuel	28
	House Coal	1
	Bottled LPG	1
EST_SPACE_HEATING_FUEL	Mains Gas	98389
	Electricity	4192
	Heating Oil	2352
	Manufactured Smokeless Fuel	164
	Solid Multi-Fuel	115
	House Coal	3
	Bottled LPG	1
EST_PREDOMINANT_ROOF_TYPE	Pitch Roof-Insulation Ceiling	102305
	Other	8432
	Flat Roof	1175
	Pitch Roof- Insulation Rafter	892
	Room in Roof- Insulation side	502
RADON_RISK	LOW	98395
	MEDIUM	10602
	HIGH	3201

In addition, Table 3 summarize numerical features, including geometric and non-geometric aspects, displaying counts, mean, standard deviation, minimum, first quartile, median, third quartile, and maximum values.

**Table 3:** Statistics of the BER dataset, numerical features.

Feature	Count	Mean	Std	Min	25 %	50 %	75 %	Max
BUILDING_HEIGHT	106484	8.2	2.13	3.02	7.33	7.89	8.56	25.87
GROUND_HEIGHT	109179	35.96	25.92	0.36	17.49	32.34	48.29	356.01
AREA	109289	90.08	119.9	5.36	46.67	58.86	86	2535.63
BER_ENERGY_MEDIAN	113195	269.6	61.23	39.8	233.8	265.4	298.6	656.6
BER_RATING_HIGH	113195	37.84	16.4	0	27	36	47	100
BER_RATING_LOW	113195	62.26	16.39	0	53	64	73	100

### 3.3 Tailte Éireann Surveying PRIME2 Dataset

**Fig. 1:**
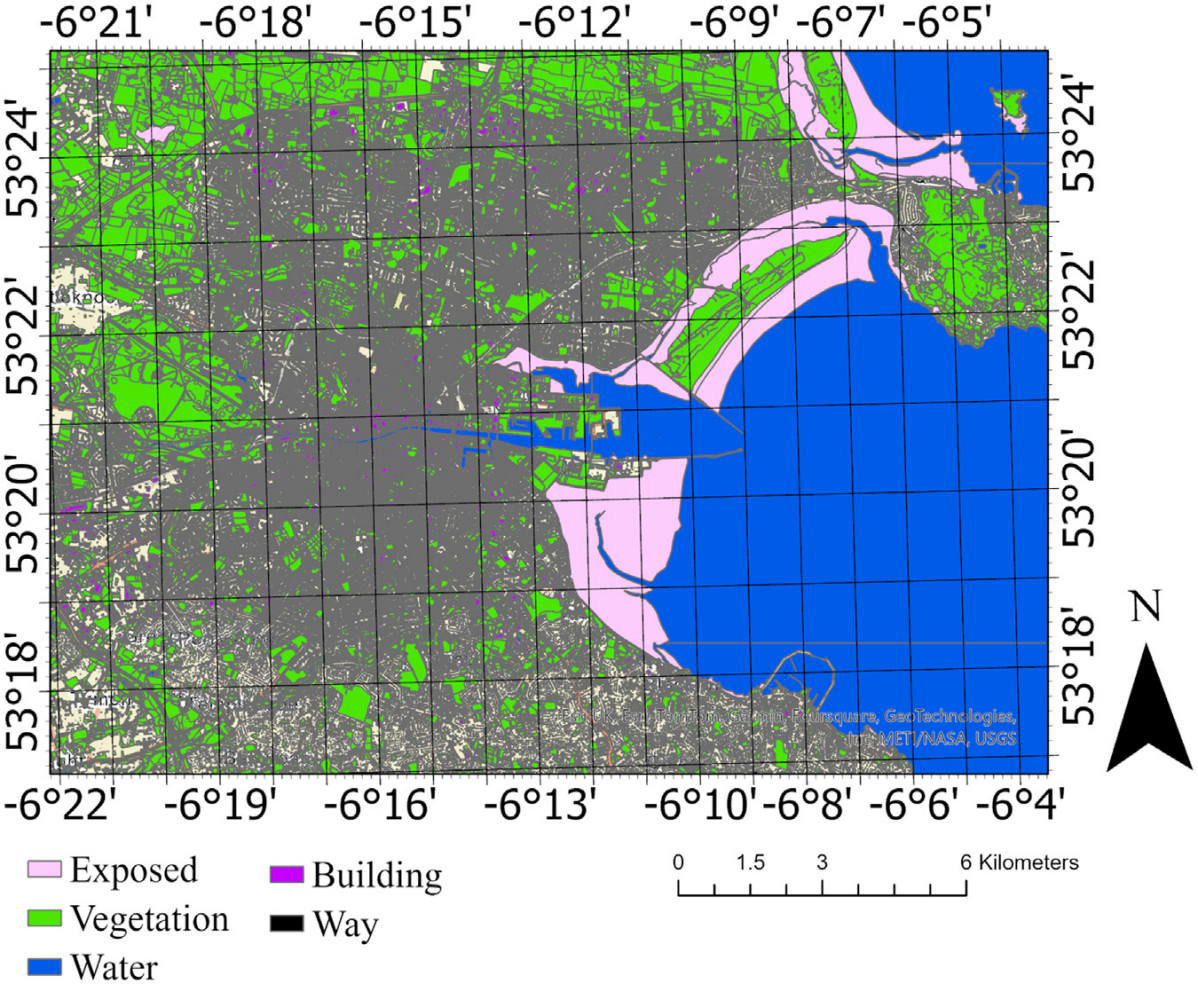
The LUCL Dublin as derived from the PRIME 2 dataset.

To effectively capture neighborhood-scale urban data, including the contextual coverage of each individual building, we must draw from various data resources. This includes accessing datasets such as Land Use Land Cover (LULC) maps and employing advanced analysistechniques like data mining algorithms. These methods are instrumental in extracting detailed information about the built environment at the neighborhood scale. Leveraging resources like the LULC map from DLM Core Data, Tailte Éireann Surveying and the PRIME2 Dataset, facilitates this endeavor, allowing us to gain comprehensive insights into the surrounding urban context. Fig. 1shows the LULC of Dublin, as depicted in the PRIME 2 dataset, illustrates the diverse land use categories and cover types across the city. This figure provides a detailed spatial representation of various land cover types within Dublin, including built environments, green spaces, water bodies, and transportation networks.

In addition, Table 4 shows the statistics of the PRIME 2 dataset.

**Table 4:** Statistics of the Tailte Éireann Surveying PRIME2 dataset.

Feature	Count	Mean	Std	Min	25 %	50 %	75 %	Max
Water	2584	175682.6	3801008	0.8	57.5	215.2	648.5	1E+08
Building	303187	84.9	365.8	0.3	21.75533	49.1	76.0	43507.72
Way	37259	783.1	1560.3	0.4	184.5619	458.9	874.7	48325.16
Vegetation	54213	2351.4	15044.7	0.3	35.4217	131.4	653.1	1094654

Ordnance Survey Ireland (OSI) introduced PRIME2, an Oracle Spatial and Graph-based object-oriented spatial data storage model. This system accommodates an extensive dataset comprising over 45 million spatial objects, covering road segments, fences, and buildings. Of these, 3,532,263 are building objects, each characterized by geodetic coordinates and a 2D polygon outlining its footprint, alongside details like form, function, and life cycle stage.

PRIME2 signifies a pivotal shift for OSI, transitioning from a conventional map-centric model to an object-oriented framework, facilitating diverse mapping and data services. It serves as a rich repository of spatial data, encompassing a range of objects including road segments, buildings, and fences. In addition to storing GIS data like polygon footprints and geodetic coordinates, PRIME2 captures supplementary building information such as address, form, function, and historical changes. This comprehensive dataset fulfils key requirements for open BIM, addressing challenges related to managing multiple building representations and seamlessly integrating authoritative geospatial datasets [6].

## 4 Experimental Design, Materials and Methods

The processed and analysismethods used in data acquisition were rooted in geoprocessing and geo-analysis of spatial data from various resources. These methodologies facilitated a deeper understanding of the surroundings encompassing each building, highlighting the importance of the characteristics of adjacent objects and the context in which each structure is situated. This comprehensive analysis extended to the microclimate conditions and energy patterns of buildings, providing valuable insights into the environmental impacts on energy needs and profiles of buildings.

We utilized the BER dataset of Ireland, obtained from GeoDirectory data, as a foundational resource. This dataset provides an wide range of building parameters, including address details, geocode data (latitude and longitude), area, height, number of floors, roof type, construction age, Radon emission, HVAC system specifications, and counts of bedrooms and bathrooms. In addition, we conducted calculations and analyses to acquire additional features, such as building orientation, and neighborhood land cover characteristics, leveraging the base dataset from GeoDirectory.

Moreover, we used the Tailte Éireann Surveying PRIME2 dataset by deriving supplementary features through map reprocessing and calculations. These enhancements enable us to extract urban-scale characteristics such as built environment density and neighbor compactness, enriching our understanding of the surrounding environment. Data collection was conducted using various methods, approaches, tools, and software, geoprocess, analysis, statistical approach and programming. Statistical part of the process and programming in Matlab, Python in Jupyter Notebook interface, using of libraries such as Geopandas, Pandas, NumPy, Seaborn, and Scikit-learn. Additionally, geospatial analyses were performed using ArcGIS and QGIS.

Given that the polygon dataset covered a smaller area than the BER dataset, we used ArcGIS Pro to search for the overlap between building polygons and the BER dataset. We used QGIS to calculate the orientation of the buildings according to the longest side of each geometry.

This approach enabled us to understand of building energy characteristics by considering buildings not only in isolation but also within their contextual environment. By adopting this comprehensive methodology, our aim was to enhance urban energy analysis by capturing the intricate relationships between buildings and their surroundings, thus facilitating more effective energy analysis at the urban scale.

**Fig. 2:**
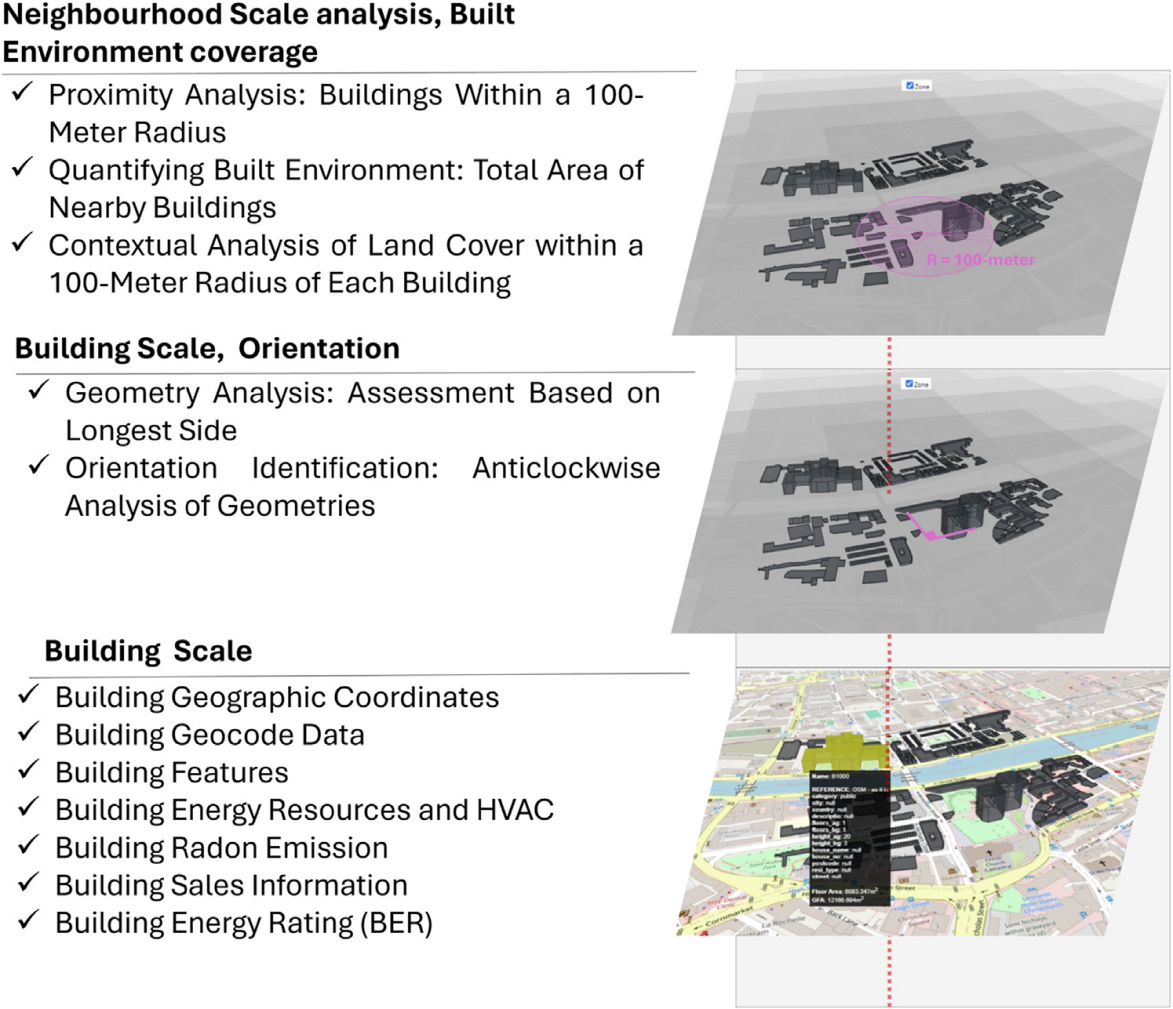
The data acquisition levels.

Fig. 2 illustrates the data acquisition framework and its two-tiered approach, utilizing two distinct data resources.

### 4.1 Case Study

**Fig. 3:**
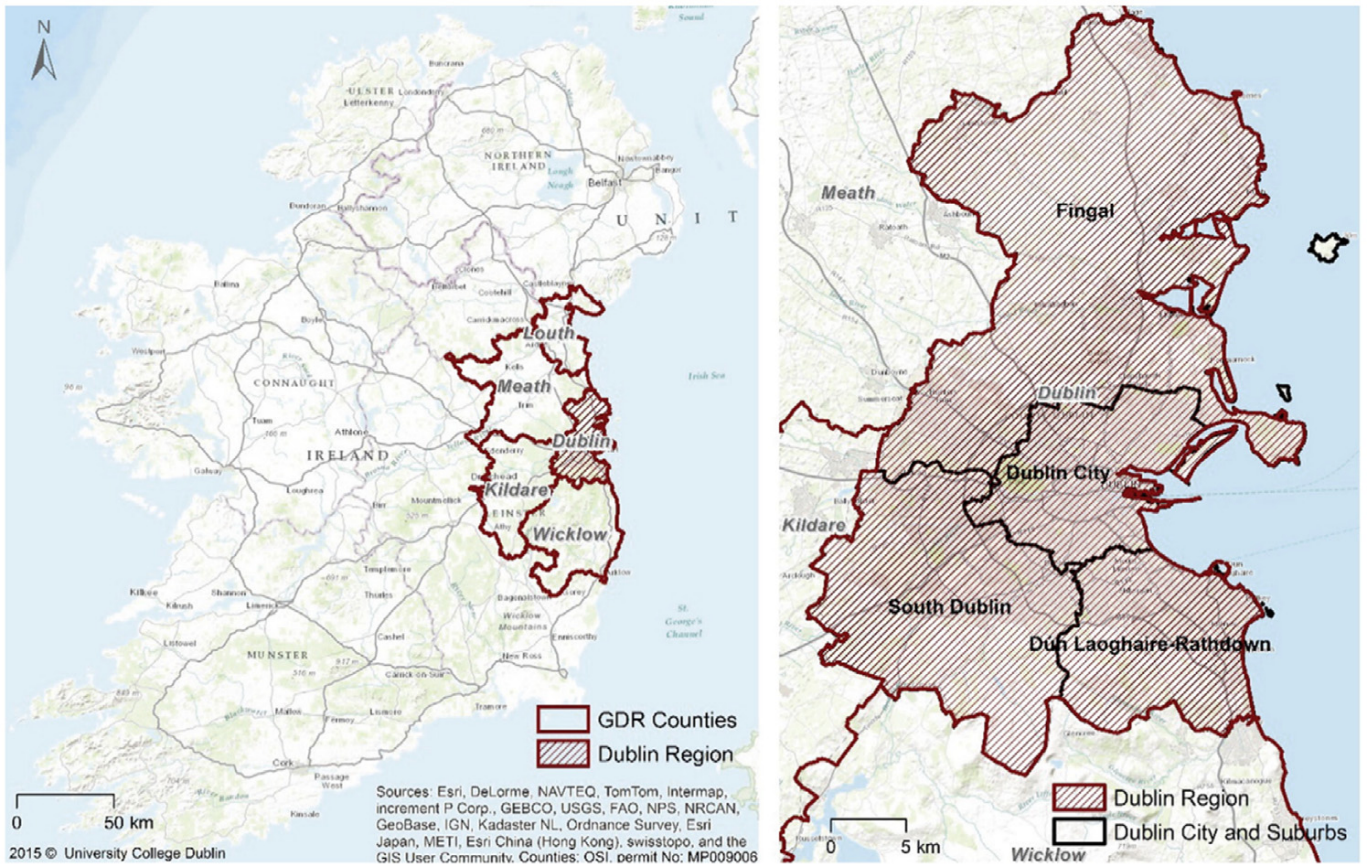
Study area of Dublin city, Greater Dublin Region (GDR) in the context of Ireland and Dublin Region [11].

Dublin, situated at 53.5°N and 6.5°W, serves as the capital of the Republic of Ireland and is among the westernmost cities in Europe [7]. As shown in Fig. 3, Dublin is positioned on the east coast, bordered by the Irish Sea to the East and the Dublin/Wicklow mountains to the South. The city predominantly occupies a flat and low-lying basin, except for the mountainous southern region [8].

Dublin covers an area of 929.4 km² (229,655.2 acres or 358.8 square miles) and has a total water area of 4,684,779.4 m², which is 0.5 % of the total area [9].

**Fig. 4:**
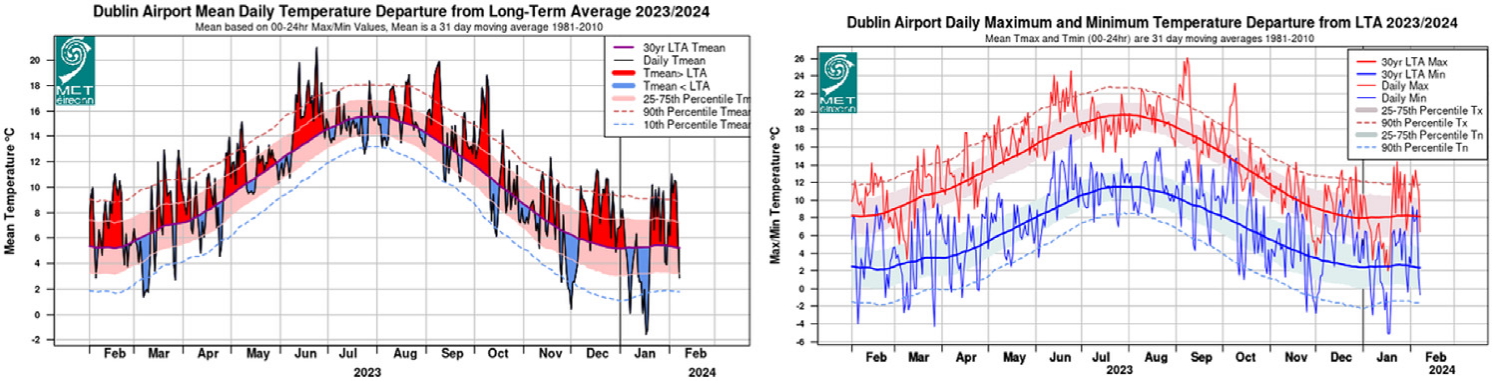
Mean daily temperature of Dublin (2023–2024) [9].

The average yearly air temperature in Ireland from 1991 to 2020 is 9.8 °C, with an annual mean ranging between approximately 8.5 °C and 10.8 °C. Coastal regions typically enjoy milder temperatures due to the tempering effect of the nearby sea, whereas elevated areas often exhibit cooler conditions. Fig. 4 depicts the average daily temperatures recorded in Dublin during the years 2023–2024, sourced from weather data collected at Dublin Airport [9].

**Geospatial Analysis for Extracting Building and Neighborhood Scale Features**

To clarify the experimental methodologies employed in obtaining data, we initially delineate the procedure for the building scale. This involves ascertaining the orientation of each individual building. Following this, we expound upon the methodologies utilized to identify features pertinent to the neighborhood scale.

1. **Building Orientation**

The orientation of each building is determined by the direction of its longer side. Conventionally, cardinal axes represent the directions, with the east direction (positive x-axis) considered as 0 degrees. Angles are calculated counterclockwise, where north corresponds to 90° and west to 180°. The building's orientation refers to the direction or angle to which its length is facing. Given that a building's axis aligns with its length, or inversely, is perpendicular to its orientation, the principal angle of the building depicted on the right side of the following figure is 45° [10].

The orientation of each building is calculated using QGIS Tool [11] from the geometry data of buildings in the BER dataset.

The expression is accessible within the field calculator window. To calculate the principal angle for all buildings in the dataset, all geometries must be referenced using $geometry within the expression section of the QGIS Tools. Two expressions can be utilized:

**Table:** 

main_angle($geometry).

2. **The Density of Built Environment**

The density of urban areas significantly influences the energy efficiency of buildings, with city compactness, determined by building proximity, serving as a crucial factor. Higher built environment density generally correlates with reduced overall energy consumption within urban areas [8]. This relationship is particularly pronounced in developing regions, where urban density plays a significant role in shaping building energy usage. Therefore, accounting for the urban context surrounding individual buildings is essential for understanding their energy patterns. Consequently, it is imperative to incorporate data on the density and compactness of the built environment, as well as the quality of land coverages, into the building energy rating dataset.

We delved deeper into the overall density of the built environment surrounding each building. This involved analyzing both the count of neighboring buildings within a 100 m radius and the cumulative area they occupy. Further, we extended this approach to analyze the land cover type surrounding each individual building. This comprehensive method enabled us to capture both the quantity and spatial distribution of nearby structures, as well as the quality of land coverage within the urban areas where the buildings are situated at the neighborhood scale.

**Fig. 5:**
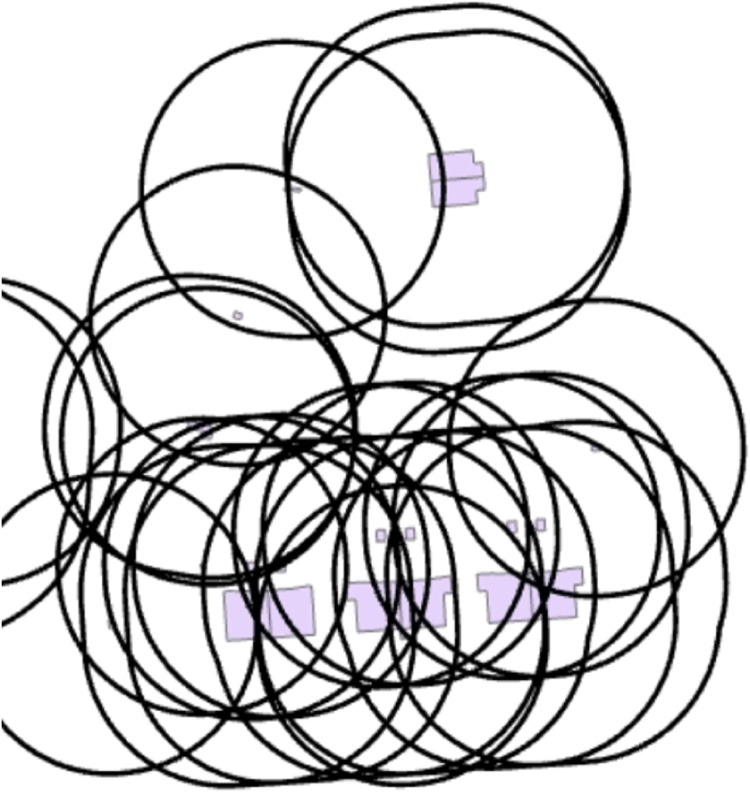
Approach to analyze the neighborhood within a 100 m radius using built environment analysis.

To assess the built environment density within a specified radius around each building, we initially experimented with calculating a 100 m buffer zone around each building's geometry as Fig. 5 shows. We then computed the total area of buildings within this buffer zone for each building. However, due to the large dataset of a major city, this approach proved excessively time-consuming and impractical, even with powerful server computers. Consequently, we developed an data analysismethod to streamline the model and simplify the calculation process. Our objective was to analyze the built environment within a 100 m radius around each building in a more efficient manner that could be executed on standard computers within a reasonable computation time, defined as completing the necessary calculations within a few hours on standard desktop or laptop computers, ensuring suitability for typical research and urban planning applications.

**First approach:** In the first approach, we used GeoPandas, Sklearn, and Shapely in Python, in Jupyter notebook interface.

This method snippet calculates the density of nearby buildings within a specified radius (e.g., 100 m) from the center of each building (Fig. 4). It uses a BallTree for efficient nearest neighbor search based on the centroids of the buildings. Finally, it adds the density information as a new column called 'Building_Density' to the shapefile dataset. The method is divided into four following steps:

1. Calculate the distance from each building to all other buildings.
2. Count the number of nearby buildings within the specified radius.
3. Assign the density value to each building.
4. Add the density information to the shapefile dataset.

This method can be implemented using GeoPandas and Shapely as follows.

Desc riptions of the codes:

**Table:** 

*# Create a BallTree using the centroids of buildings for efficient nearest neighbor search*
centroids = gdf.geometry.centroid
tree = BallTree(centroids.apply(lambda x: [x.x, x.y]).tolist(), leaf_size=40)
*# Define the radius for density calculation (e.g., 100 m)*
radius = 100
*# Calculate the density for each building*
densities = []
for i, centroid in enumerate(centroids):
*# Query the BallTree to find all neighbors within the radius*
neighbors = tree.query_radius([[centroid.x, centroid.y]], r=radius)[0]
*# Subtract 1 to exclude the building itself*
density = len(neighbors) - 1
densities.append(density)
*# Add the density information to the GeoDataFrame*
gdf['Building_Density'] = densities
*# Save the updated GeoDataFrame to a new shapefile*
gdf.to_file('path_to_output_shapefile.shp')

**Second approach:** In this approach, our goal was to assess the density of nearby buildings within a designated radius of 100 m from each building's center. We needed a method that could handle large-scale data analysis efficiently across the entire city without consuming excessive time. To accomplish this, we utilized MATLAB to create a 100 m buffer around the geometry of each building. We then systematically processed each building, calculating the total area covered by buildings within this buffer zone. This approach provided us with a comprehensive overview of the density of neighboring structures relative to each building.

Furthermore, we expanded our analysis to include land cover type surrounding each building. This involved enriching the dataset with additional information on the composition of the built environment. We quantified various components such as the proportion of area occupied by buildings, green spaces, water bodies, pathways, and other surface types. By integrating this data, we gained valuable insights into the distribution and makeup of the urban environment surrounding each building, thereby enhancing our understanding of neighborhood-scale built environment density.

Descriptions of the codes:

**Table:** 

*#To convert geographical coordinates (latitude and longitude) to coordinates on the UTM (Universal Transverse Mercator) projection, for distance extraction.*
[x,y,utmzone] = UTM(instruccion)
*#To adjust and resize the image to match the dimensions of the reference image, maintaining the original content. Add or remove rows and columns as needed to ensure compatibility for saving in GeoTIFF format.*
function Imout=Imresize(im1,im2)
*#Conver Im1 (Original) to Im2 (Resized) image*
[n1,m1]=size(im1);[n2,m2]=size(im2);
if (n1>=n2 && m1>=m2)
Imout=im1(1:n2,1:m2);
elseif (n1<=n2 && m1>=m2)
Imout=zeros(n2,m2);
Imout(1:n1,:)=im1(:,1:m2);
elseif (n1<=n2 && m1<=m2)
Imout=zeros(n2,m2);
Imout(1:n1,1:m1)=im1;
else
disp('Error')
end
*#The main program, Imresample_analysis.m, is responsible for calling the converted polygon format images to raster. It processes these images to determine their numerical values as a percentage in the desired scale.*
function Resize_Write_Raster(im_name,new_resolution)
[Z,R] = readgeoraster(im_name);Z=double(Z);
info = geotiffinfo(im_name);
Z(Z==65535)=0;Z(Z>0)=1;Z(Z<0)=0;
*#Define new resolution*
[Z2,R2] = mapresize(Z,R,1/new_resolution,'nearest');
*#Calculate resampling factor*
resampling_factor = new_resolution / info.PixelScale(1);
*#Perform resampling and aggregation*
resampled_raster = blockproc(Z, [resampling_factor resampling_factor], ...
@(block_struct) sum(block_struct.data(:)));
resampled_raster=Imresize(resampled_raster,Z2);
resampled_raster=floor((resampled_raster/(new_resolution^2))*100);
*#Write the resampled raster to a new geotiff file*
geotiffwrite(strcat(im_name(1:end-4),'_Resample_percentage.tif'), resampled_raster, R2,'GeoKeyDirectoryTag', info.GeoTIFFTags.GeoKeyDirectoryTag);
***#****The main function of the program*

**Radious_calculate_building_complexity Program** *prompts the user to input the name of the image and the desired scale. Subsequently, the program calculates the percentage of pixels with values greater than 0 in the input image. This information is then utilized to determine the percentage of pixels in the new image at the specified scale* **.**

**Table:** 

clc;clear;
My_dist=100;
proj = projcrs(32629);
tb_ber=readtable('Dublin, residetial buildings09052024_insideBBox.csv');
tb_ber(:,1:25)=[];
Lat_ber=tb_ber.LATITUDE;Lon_ber=tb_ber.LONGITUDE;
[x_ber,y_ber] = projfwd(proj,Lat_ber,Lon_ber);
tb_poly=readtable('Building_poly_centriod.csv');
area_poly=tb_poly.SHAPE_Area;
Lat_poly=tb_poly.Y_center;Lon_poly=tb_poly.X_center;
[x_poly,y_poly] = projfwd(proj,Lat_poly,Lon_poly);
m=10000;
tic
n_ber=length(x_ber);n_poly=length(x_poly);
index_subset_ber_start=1:m:n_ber;index_subset_ber_start(end)=[];
index_subset_ber_end=0:m:n_ber;index_subset_ber_end(1)=[];
index_subset_ber_end(end)=n_ber;
U_area_ber=zeros(size(x_ber));
for k=1:length(index_subset_ber_end)
id_bersub=index_subset_ber_start(k):index_subset_ber_end(k);
xber_sub=x_ber(id_bersub); yber_sub=y_ber(id_bersub);
mk=length(xber_sub); N=n_poly*mk;
*#BER Vector Construction*
tic
Xber=repmat(xber_sub,1,n_poly);Xber=Xber';Xber=Xber(:);
Yber=repmat(yber_sub,1,n_poly);Yber=Yber';Yber=Yber(:);
Xpoly=repmat(x_poly,1,mk);Xpoly=Xpoly(:);
Ypoly=repmat(y_poly,1,mk);Ypoly=Ypoly(:);
Di=((Xber-Xpoly).^2+(Yber-Ypoly).^2).^0.5;
toc
tic
Diii=zeros(10000,416900);
for j1=1:10000
for j2=1:416900
Diii(j1,j2)=((xber_sub(j1)-x_poly(j2))^2+(yber_sub(j1)-y_poly(j2))^2)^0.5;
end
end
toc
Area_poly=repmat(area_poly,1,mk);Area_poly=Area_poly(:);
Id=find(Di>My_dist);Area_poly(Id)=0;
Area_poly=reshape(Area_poly,n_poly,mk);
U_area_ber(id_bersub)=sum(Area_poly,'omitmissing');
end
tb_ber.Ur_complx=U_area_ber;
toc
#s Elapsed time for the Matrix based computation is 549.582201 s.
writetable(tb_ber,'BER_Residential_index.csv')

In the program Radious_calculate_building_complexity.m, written based on dot product techniques, we calculated the distances between points of one point layer (the centers of polygons) with another point layer (BER dataset). This method allows simultaneous analysis of a large set of points without writing a loop for each point, depending on the system's RAM memory. For example, in we simultaneously calculated the distance of 10,000 points to another point layer containing 416,900 points. If it were written with a loop, the program would have to repeat the loop 10,000 times, requiring a significant amount of time. Even in some other programming languages, this operation needs to be repeated in two separate nested loops, one 10,000 times and the other 416,900 times, to calculate all distances between the points of the two sets. In contrast, using the dot product method, this process took only 18.101 s, compared to 82.745 s with loops, making it approximately 4.55 times faster. The reasons for the fast execution of matrix calculations in dot product form compared to nested loops for calculation in MATLAB can be attributed to the following:

The dot product, also known as the scalar product or inner product, is a fundamental operation in linear algebra. In the context of MATLAB, the dot product is often used to compute the sum of the products of corresponding entries of two vectors.

**Vectorization**: MATLAB is optimized for matrix and vector operations. When you perform the dot product using MATLAB's built-in functions, it leverages highly optimized linear algebra libraries like BLAS (Basic Linear Algebra Subprograms) and LAPACK (Linear Algebra Package) under the hood. These libraries are written in low-level languages like Fortran or C, and they are highly optimized for performance.

**Cache Efficiency**: Modern CPUs have cache memories that store frequently accessed data. MATLAB's dot product implementation is optimized to take advantage of CPU caches, reducing the need to fetch data from slower main memory.

**Parallelization**: Depending on the size of the vectors and the hardware configuration, MATLAB may parallelize the dot product computation across multiple CPU cores, further enhancing performance.

**Compiler Optimizations**: MATLAB's JIT (Just-In-Time) compiler can optimize the dot product computation for the specific architecture on which it is running. This includes loop unrolling, instruction scheduling, and other optimizations that improve performance.

**Precompiled Libraries:** MATLAB comes with precompiled BLAS and LAPACK libraries that are highly optimized for various hardware platforms. These libraries are tuned to extract maximum performance from the underlying hardware.

By utilizing these advanced techniques and tools, we efficiently calculated the density of buildings within the designated radius and enriched our dataset with comprehensive neighborhood-scale built environment metrics.

## 5 Final Building Energy Dataset

After carefully considering various data acquisition methods to capture building-scale and neighborhood-scale features, including both geometric and non-geometric attributes, as well as information about the surrounding built environment, we compiled a comprehensive dataset. This dataset encompasses all relevant information associated with each building's unique ID. Due to data protection policies, spatial details such as latitude, longitude, and addresses were omitted. However, the remaining data, aligned with our data acquisition objectives, was structured into a CSV file, available in the mentioned repository.

The dataset incorporates information at two scales: building and neighborhood. The building-scale features include attributes such as building form, function, dimensions, energy ratings, and structural details. On the other hand, neighborhood-scale features encompass characteristics like soil composition, water bodies, built-up areas, green spaces, road networks, and other land uses.

Building-scale features include attributes such as Building ID, Form, Function, Shape Length, Shape Area, Building Main Angle, Building Height, Ground Height, Building Area, Number of Floors, Unit Use, Building Use, Building Type, Property Size, Small Area, BER (Building Energy Rating) Energy, BER Rating, BER Ratio 1, BER Ratio 2, Year of Built, Estimated Bedrooms, Estimated Bathrooms, Landslide Risk, Estimated Structure, Estimated Water Heating Source, Estimated Space Heating Source, Estimated Roof Type, and Radon Risk.

Neighborhood-scale features encompass Soil Group, Soil Definition, Waterbody Value, Built Area Value, Greenbody Value, Roads and Ways Value, and Other Spaces Value. Table 5 presents the features of the generated dataset along with their original sources.

**Table 5:** Features of the created dataset and their sources.

Feature	Original data	Analysed/reproduced data	Resources	
			BER dataset	PRIME2 Dataset
**Building scale**				
BUILDING_ID	*		*	
FORM_VALUE	*		*	
FUNC_VALUE	*		*	
SHAPE_LENGHT	*		*	
SHAPE_AREA	*		*	
BUILDING_MAIN_ANGLE		*	*	
BUILDING_HEIGHT	*		*	
GROUND_HEIGHT	*		*	
BUILDING_AREA	*	*	*	
FLOORS	*		*	
UNIT_USE	*		*	
BUILDING_USE	*		*	
BUILDING_TYPE	*		*	
PROPERTY_S	*		*	
SMALL_AREA	*		*	
BER_ENERGY	*		*	
BER_RATING	*		*	
BER_RATI_1	*		*	
BER_RATI_2	*		*	
YEAR_OF_BULT	*		*	
EST_BEDROOMS	*		*	
EST_BATHROOMS	*		*	
LANDSLIDE	*		*	
EST_STRUCTURE	*		*	
EST_WATER_HEATING_SOURCE	*		*	
EST_POOFTYPE	*		*	
RADON_RISK	*		*	
**Neighborhood scale**				
SOIL_GROUP		*		*
SOIL_DEFINITION		*		*
WATERBODY_VALUE		*		*
BUILTAREA_VALUE		*		*
GREENBODY_VALUE		*		*
ROADS, WAYS_VALUE		*		*
OTHERSPACES-VALUE		*		*

By integrating data from diverse sources using innovative methods, our dataset offers valuable insights into building energy performance and urban dynamics. Researchers can utilize this data to develop data-driven approaches and predictive models for analyzing environmental factors and devising sustainable urban planning strategies. This dataset serves as a valuable resource for studying energy analysis, sustainability, and urban development in Dublin's residential areas and neighborhoods.

## Limitations

The limitations associated with data availability and the scope of analysis are pivotal in this study. Our reliance on the GeoDirectory dataset and PRIME 2 database introduces potential variability in data accuracy and completeness, thereby impacting the reliability of our findings. Moreover, the sheer size of the initial dataset presents challenges in conducting a thorough analysis. As a result, we focused our attention specifically on buildings within Dublin, rather than encompassing all buildings across Ireland. Additionally, due to incomplete coverage of certain polygon layers in the original data, we were compelled to classify certain areas as "other spaces" in our analysis.

While this targeted approach enables a more manageable case study, it runs the risk of overlooking other pertinent factors that could influence energy efficiency in urban environments beyond residential settings.

## Ethics Statement

The authors have read and follow the ethical requirements for publication in Data in Brief and confirming that the current work does not involve human subjects, animal experiments, or any data collected from social media platforms.

## CRediT authorship contribution statement

**Nasim Eslamirad:** Conceptualization, Methodology, Data curation, Writing – original draft, Writing – review & editing, Formal analysis. **Mehdi Gholamnia:** Methodology, Data curation, Writing – review & editing. **Payam Sajadi:** Writing – review & editing. **Francesco Pilla:** Supervision, Funding acquisition, Project administration.

## Data Availability

Building and Neighborhood Data Considering Energy in Dublin, Ireland (Original data) (Mendeley Data).
